# Use of ß-blocker therapy to prevent primary bleeding of esophageal varices

**DOI:** 10.1111/j.1745-7599.2010.00567.x

**Published:** 2010-12

**Authors:** Tammy Tursi

**Affiliations:** Endoscopy Department, Hospital of the University of PennsylvaniaPhiladelphia, Pennsylvania

**Keywords:** Beta-blocker, hepatology, gastroenterology, patient education

## Abstract

**Purpose:** The purpose of this article is to educate nurse practitioners about the pathophysiology surrounding the development of portal hypertension and the effective use of nonselective ß-blockers to prevent primary bleeding and decrease the mortality risk.

**Data sources:** The articles included were retrieved via ISI Web of Science using the years 2004–2009 and key words cirrhosis, portal hypertension, esophageal varices, and beta-blockers. This information included scholarly books, journal reviews, retrospective chart reviews, and prospective randomized studies.

**Conclusions:** Cirrhosis is the leading cause of portal hypertension in Europe and North America. Esophageal varices are a result of the portosystemic collaterals the body develops to decompress the portal system. Hemorrhage from esophageal varices is a major cause of morbidity and mortality. Prevention of a primary bleed is the goal of therapy and is accomplished with nonselective ß-blockers.

**Implications for practice:** Very few patients with portal hypertension and esophageal varices are on ß-blockers. Use of nonselective ß-blockers has been found to lower portal pressure and decreases the risk of bleeding from esophageal varices and therefore decreases mortality. Patients unable to use ß-blockers can undergo endoscopic variceal ligation as an alternate method to reduce risk of bleeding.

Patients with portal hypertension are at an increased risk of developing esophageal varices. In Europe and North America, cirrhosis accounts for 90% of the cases of portal hypertension (Triantos & Burroughs, 2007). Other causes can include thrombosis of any of the veins leading to the liver (portal, superior mesenteric, or splenic), idiopathic portal hypertension, primary biliary cirrhosis, primary sclerosing cholangitis, vitamin A toxicity, infiltrative disorders, veno-occlusive disease, Budd-Chiari syndrome, and congestive heart failure (Toubia & Sanyal, 2008).

Esophageal varices are a major concern in patients with cirrhosis. These varices have a high propensity to bleed because of the fragility of the engorged blood vessels (Cichoz-Lach, Celinski, Slomka & Kasztelan-Szczerbinska, 2008). Each episode of bleeding has a 30%–50% mortality risk. Furthermore, after the initial episode of bleeding the incidence of rebleeding is up to 70% and frequently occurs within 6 weeks of the initial hemorrhage (Sharma & Rakela, 2005; Toubia & Sanyal, 2008).

For patients with esophageal varices the goal of treatment is to reduce portal hypertension. By reducing portal pressure there is a concomitant decrease in the probability of esophageal bleeding. Reducing the risk of primary bleeding of esophageal varices can be accomplished by using nonselective ß-blocker therapy. This pharmacological intervention works by reducing the hepatic venous pressure gradient (HVPG). The HPVG is a measure of the pressure difference in the portal vein and the intra-abdominal portion of inferior vena cava and is typically between 5 and 10 mmHg (Toubia & Sanyal, 2008).

Unfortunately, many patients with portal hypertension are not receiving ß-blocker therapy or are not on doses adequate to attain therapeutic results. The reasons include: therapy is never initiated, the dose is not therapeutic, or the patient or the healthcare provider discontinues ß-blockers because of the side effects.

Even with treatment of nonselective ß-blocker therapy only 38% of patients respond (Sharma, Kumar, Sharma & Sarin, 2009). At present there is no way to identify who will be a responder unless the invasive method of HVPG measurement is used. Instead, adequate endpoints of ß-blocker therapy are monitored noninvasively by heart rate and blood pressure reduction. While this is not always an indication of being a responder, it is easy, noninvasive, and the most accepted method to date.

In spite of the documented need to reduce heart rate by 20%–25% with ß-blocker therapy, healthcare providers are not reaching this endpoint or just not prescribing the medications. There is a knowledge deficit related to the need to use this pharmacological intervention. Therefore, this is an area in which nurse practitioners (NPs) can make great strides. By starting the patient on ß-blocker therapy and monitoring closely for side effects, NPs can adjust dosages and provide encouragement for patients to “stay the course” and let their body adapt to the treatment regimen.

## Pathophysiology

The normal HVPG is typically 5–10 mmHg. The risk of developing esophageal varices increases when the HVPG reaches a minimum pressure of 10–12 mmHg (Toubia & Sanyal, 2008). Portal hypertension, as measured by the HVPG, occurs through complex physiological responses resulting in both mechanical and dynamic blockages in the liver (Dib et al., 2006). The mechanical component of blockage is a result of intrahepatic fibrosis starting at the level of hepatic microcirculation. The fibrosis develops because of damage to hepatocytes occurring as a result of alcohol or other liver-damaging processes. Fibroblast and activated hepatic stellate cells appear at the area of injury and deposit collagen. This process leads to connective tissue formation in the periportal and pericentral zones of the liver. These spiderweb-like tissue formations eventually connect the portal triads and central veins of the liver. Also, these areas of fine connective tissue surround small groups of liver cells and, in turn, cause regeneration of the liver cells with resulting nodule formation. This mechanical process causes hardening of the tissue and nodule formation within the liver and initiates the blockage of circulatory pathways (Kasper et al., 2005).

The second component involved in portal hypertension is dynamic blockage. This process occurs as a result of inappropriate vasoconstriction and vasodilation within the portal system. Vasoconstriction occurs because of the inappropriate contraction of portal and septal myofibroblasts, hepatic stellate cells, and vascular smooth muscle cells. This takes place through an assortment of vasoconstrictors being activated when damage occurs to the hepatocytes. The vasoconstrictors involved include norepinephrine, endothelin, angiotension II, leukotrienes, and thromboxane A2 (Dib et al., 2006). As these vasoconstrictors become both overproductive and hyperresponsive as a result of the damage to the hepatocytes, pressure within the portal system increases to levels that can induce the development of esophageal varices (Cichoz-Lach et al., 2008). This dynamic component is further compounded by the activation of vasodilators, such as nitric oxide, which increase the portal vascular congestion through increased blood flow. This portion of the hyperkinetic circulation develops because of the nitric oxide increasing the cardiac output and promoting pervasive vasodilation of the vascular bed. Finally, as a result of the increased dynamic blood flow there is constant shear stress in the vascular structures that leads to vascular remodeling. The mechanical and dynamic blockage processes lead to a continuing cycle that contributes to the increase in portal hypertension (Cichoz-Lach et al., 2008).

The sequela of this entire process is esophageal varices. Varices form as a result of the body's effort to decompress the portal vein and return blood to the systemic circulatory system. The body achieves this through development of portosystemic venous collaterals that can form at many sites. These sites include the rectum, umbilicus, retroperitoneum, and the distal esophagus and proximal stomach. While other sites of collateral circulation can develop, most patients will develop gastroesophageal varices because this allows for the largest collateral flow via the short and left gastric veins (Toubia & Sanyal, 2008). In fact, of patients with portal hypertension an overwhelming majority, approximately 80%–90%, will develop gastroesophageal varices (Cichoz-Lach et al., 2008).

## Diagnosis

Patients suspected of having cirrhosis are definitively diagnosed by liver biopsy. This is considered the gold standard for determining the presence and severity of necroinflammatory activity and fibrosis (Thuluvath & Krok, 2005). After confirming cirrhosis, the best method to identify esophageal varices is via upper endoscopy. This technique provides direct visualization of the esophagus and stomach and, if varices are present, the ability to determine their size and appearance. It also allows for interventional treatment such as endoscopic variceal ligation (EVL) if the varices are large and the patient is not a candidate for ß-blocker therapy (Toubia & Sanyal, 2008; Triantos & Burroughs, 2007).

**Figure 1:**
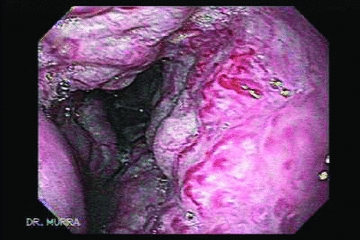
Variceal appearance on endoscopy ("red signs"): red wale marks (longitudinal red streaks on varices); cherry-red spots (red, discrete, flat spots on varices); hematocystic spots (red, discrete, raised spots); diffuse erythema (Murra-Saca, 2009).

**Figure 2:**
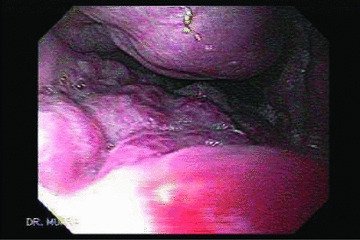
Esophageal varices. Example of esophageal varices (Murra-Saca, 2009).

At present, upper endoscopy is the gold standard to diagnose varices. In the future, a multidetector computed tomography (CT) esophagography could be an option to evaluate for varices in a less invasive manner. The major drawbacks to CT at this time include lack of ability to perform therapeutic intervention if needed at the time of evaluation, inability to assess the qualitative endoscopic appearance, such as red signs (Figures 1 and 2), and an increased risk of radiation exposure as compared to routine CT examination (Kim et al., 2007).

When esophageal varices are present it is imperative to categorize their appearance. Currently, there are two accepted methods to describe varices. One method is that of the Japanese Research Society for Portal Hypertension, which describes them by red color signs, the color of the varix, the form (size), and the location (Toubia & Sanyal, 2008). The second method is described by the Northern Italian Endoscopy Club and identifies them as F1, F2, or F3, with this identification system corresponding with small, medium, and large. It also identifies any red marks on the varices (Toubia & Sanyal, 2008). Whichever method is used, it is important to understand the basic premise that the larger the varices the greater the propensity to bleed.

Both methods provide for the recognition of red marks. The red sign can be described as red wale marks that are longitudinal red streaks on the varix. Cherry red spots are another common occurrence on varices. These are red, discrete, flat spots on the varices. The presence of either of these can be viewed as an ominous sign indicating an increased risk of bleeding from varices (Toubia & Sanyal, 2008).

Screening for varices should be initiated when a patient is diagnosed with cirrhosis. In cirrhotic patients there is a 5%–15% chance per year of developing varices. After varices appear, they will increase in size 4%–10% annually (Toubia & Sanyal, 2008). If the initial endoscopy reveals no varices, screening can be performed every 3 years. For those patients who have small varices, screening should be performed every 2 years. However, if large varices are found there is no need to do follow-up endoscopy. Instead, primary prophylaxis should be initiated immediately with nonselective ß-blocker therapy if there are no contraindications such as bronchial asthma, hypotension, bradycardia, 2nd or 3rd degree heart block, or chronic obstructive pulmonary disease (COPD; Dib et al., 2006). In patients with large varices who are started on ß-blockers, posttreatment endoscopy is not performed because the risk of a variceal bleed outweighs the benefit of the procedure. Repeat endoscopy would only be performed in the setting of hemorrhage from esophageal varices.

**Figure 3:**
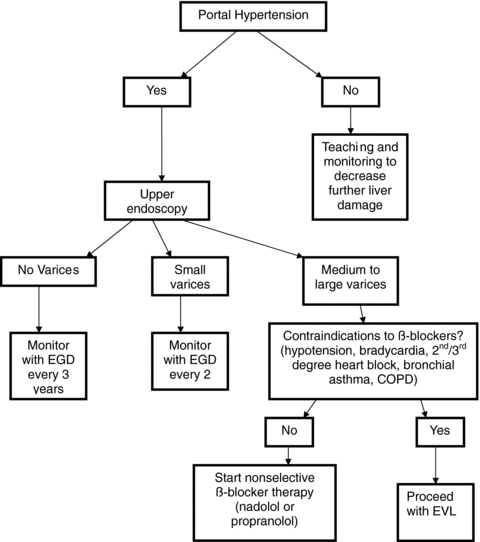
Management of varices: EGD, esophogastroduodenoscopy; EVL, endoscopic variceal ligation.

For patients with contraindications to ß-blocker therapy, or considered at high risk for noncompliance with the medication regimen, EVL would be the secondary treatment option. EVL is the second line therapy because there is a risk of bleeding from the varices postprocedure. See Figure 3 for a suggested approach to management.

It is interesting to note that studies do not show any benefit in starting ß-blocker therapy prior to the development of varices because it does not prevent them from forming. However, some studies do advocate starting ß-blocker therapy when small varices develop to help slow progression (Triantos & Burroughs, 2007). More studies need to be conducted to validate this finding.

In addition to concern about esophageal varices, it is important to note there are other sequelae of portal hypertension. There can be a rise in serum bilirubin with associated jaundice; this is a hallmark sign of end stage liver disease (ESLD). The serum albumin will also decrease in patients with portal hypertension because albumin is synthesized solely by hepatocytes. Moreover, hepatocytes are responsible for the production of blood clotting factors. As the number of functioning hepatocytes decrease, the prothrombin time elevates. Ascites can also be a physical finding in patients with portal hypertension. The cause is unclear but one theory is the sequestration of fluid in the splanchnic vascular bed leads to decreases in effective circulating volume. This triggers the kidneys to retain salt and water. The hypoalbuminemia and reduced plasma oncotic pressure then increase the incidence of extravasation of fluid into the peritoneal cavity. Another result of portal hypertension is the development of hepatic encephalopathy. While the cause is unknown, it is theorized that it is related to various toxic substances being absorbed from the intestinal tract, but not detoxified by the liver (Kasper et al., 2005).

As part of the evaluation and management of cirrhosis and portal hypertension, it is important to stage this disease process with the Child-Pugh score. This scoring method can assist in identifying those at higher risk for major complications such as esophageal varices. Calculation of a Child-Pugh score is done by assessment of serum bilirubin level, serum albumin level, prothrombin time, presence or absence of ascites, and hepatic encephalopathy (Table 1). Based on the scores, patients are placed in Category A (5–6 points), B (7–9 points), or C (10–15 points), with the higher scores indicating a more advanced state of disease and higher risk of major complications (Kasper et al., 2005).

**Table 1:** Child-Pugh score

Factor	Unit	1 point	2 points	3 points
Serum bilirubin	μmole/L	<34	34–51	>51
	mg/dL	<2.0	2.0–3.0	>3.0
Serum albumin	g/L	>35	30–35	<30
	g/dL	>3.5	3.0–3.5	<3.0
Prothrombin time	Second Prolonged INR	0–4	4–6	>6
		<1.7	1.7–2.3	>2.3
Ascites		None	Easily controlled	Poorly controlled
Hepatic encephalopathy		None	Minimal	Advanced

*Note.* Add scores from the five factors. Child-Pugh scores are: class A (score of 5–6), B (7–9), or C (10 or above). If Child-Pugh Score is 7 or more in patients with cirrhosis, it is an indication of decompensation (Kasper et al., 2005). INR = international normalized ratio.

## Management

In treating patients with esophageal varices the goal of therapy is primary prevention of bleeding. At present there are two options available. The first line treatment is pharmacological intervention with nonselective ß-blocker therapy. The second treatment option available, if there are contraindications to nonselective ß-blockers, is EVL. In using EVL it is important to note it is only used with medium to large varices because the risk of bleeding with EVL post treatment outweighs the benefit in treatment of smaller varices.

Nonselective ß-blockers have been shown to lower the HVPG. The median reduction of the HVPG with nonselective ß-blocker therapy is 15%. Some studies have presented evidence that bleeding of esophageal varices generally does not occur until the pressure gradient is more than 12 mmHg. Therefore, even a modest reduction of 15% can potentially reduce HVPG from 12 to 10.2 mmHg, thus greatly reducing the risk of bleeding (Dib et al., 2006). In fact, treatment with nonselective ß-blocker therapy shows evidence of reducing the risk of primary bleeding of esophageal varices by up to 50%. Along with decreasing the risk of bleeding comes a reduction in mortality by 25%–45% when compared to no therapeutic intervention (Thuluvath, Maheshwari, Jagannath, & Arepally, 2005). Meta-analyses also support these results by showing a 40% decrease in bleeding risk in patients with whom nonselective ß-blocker therapy is used (Wilbur & Sidhu, 2005).

Nonselective ß-blockers work by reducing portal blood flow. This occurs through decreased cardiac output and decreased azygous blood flow as a result of ß-1 receptor blockade. Vasoconstriction occurs because of unopposed alpha vasoconstriction effect which leads to arteriolar splanchnic vasoconstriction. This physiological response to the nonselective ß-blocker therapy leads to less blood flow into the portal system (Dib et al., 2006; Wilbur & Sidhu, 2005). Use of other ß-blockers, such as atenolol or metoprolol, do not cause splanchnic vasoconstriction and therefore would not reduce portal blood flow and portal pressure (Wilbur & Sidhu, 2005).

Interestingly, it has been noted by Toubia and Sanyal (2008) that bacteremia is often present in patients admitted for acute variceal hemorrhage. A study conducted by Perez-Paramo et al. (2000) examined cirrhotic rats with ascites and found that use of propranolol decreased the rate of bacterial translocation. In this study the intestinal overgrowth was associated with intestinal hypomotility. Propranolol accelerated the intestinal transit and decreased the rate of bacterial overgrowth. This led to less translocation of bacteria. This type of effect becomes especially important in human patients with ascites because it could reduce the risk of spontaneous bacterial peritonitis, another major health concern in patients with portal hypertension (Dib, Oberti & Cales, 2006). Patients at risk for spontaneous bacterial peritonitis can be identified through the Child-Pugh score.

In a study conducted by Lay et al. (2006), which examined the use of nonselective ß-blocker therapy as compared to EVL in lowering the risk of a primary bleed, the target heart rate was aimed at a 20% reduction of the resting heart rate. Their study protocol used propranolol started at 40 mg twice a day and titrated up by 10 mg twice a day to obtain the goal heart rate. Other studies comparing nonselective ß-blocker therapy and EVL, such as that of Lo et al. (2004), used nadolol with the goal of a 25% reduction in heart rate or to decrease heart rate to 55 beats per minute. Both nonselective ß-blocker therapy and EVL were effective in decreasing the incidence of hemorrhage of esophageal varices and mortality associated with hemorrhage. Lo et al. (2004) reported a bleeding rate at 2 years of 10% (EVL) and 18% (nonselective ß-blocker therapy) and a 2-year mortality rate of 22% (EVL) and 24% (nonselective ß-blocker therapy). Lay et al. (2006) found similar findings with risk of bleeding at 2 years of 19.2% (EVL) and 16.9% (nonselective ß-blocker therapy) and mortality at 2 years of 28% (EVL) and 24% (nonselective ß-blocker therapy). Whether nadolol or propranolol is used is at the discretion of the NP. The main goal is that NPs understand the importance of the medication and prescribe and monitor the medications appropriately.

Medication noncompliance with ß-blocker therapy will be a major concern with most patients. The side effects include fatigue, dizziness, impotence, dry mouth, nausea, vomiting, diarrhea, and constipation. Education will be crucial in helping patients stay on the medication. If patients understand that their body will adjust and the side effects will wane, they will be more likely to continue medication therapy.

Patient education should include instruction on how to monitor the heart rate. The easiest method for patients is to use the second and third fingers of one hand to feel for the radial pulse on the opposite wrist area. Instruct patients to count the pulse for 30 s and then multiply by 2 for their heart rate per minute. Patients should monitor their heart rate before each dose of ß-blocker medication with a target heart rate of 55 beats per minute. If their heart rate is below this rate they should hold the dose and call for further instructions. Frequent monitoring of heart rate and slow titration of the medication will allow patients time to adapt to the therapy and be more likely to continue on ß-blocker treatment.

In cases where patients are already on ß-blockers (i.e., metoprolol or carvedilol) for treatment of heart failure, the medical teams will need to decide if switching to nonselective ß-blocker therapy is a viable option. The decision must be based on which health concern, heart failure or portal hypertension, is of greater need. The ß-blockers used for heart failure will not have the desired effect of reducing portal blood flow. Likewise, the nonselective ß-blocker therapy used for portal hypertension will not have the desired effect on heart failure patients of helping to improve cardiac function and reduce mortality. When patients are on ß-blockers for other reasons than heart failure (such as hypertension, angina, arrhythmias, or myocardial infarction) there is less concern about switching medications to nonselective ß-blocker therapy because similar effects are attained.

EVL is a treatment option available for patients who are not candidates for nonselective ß-blocker therapy. Contraindications for ß-blocker therapy include bronchial asthma, hypotension, bradycardia, 2nd or 3rd degree heart block, or COPD (Dib et al., 2006; Krige, Shaw, & Bornman, 2005). Additionally, EVL is also the treatment option for patients who are noncompliant with the medication regimen or those patients who experience an esophageal variceal bleed despite ß-blocker therapy. EVL is a moderately invasive procedure that places rubber bands on esophageal varices during upper endoscopy. First the varices are identified and then a special device is used to suck the varix into a cylindrical tube and a rubber band is released to effectively strangle the varix. The varix will become necrotic and slough off, with the rubber band, over the course of a week (Krige et al., 2005). EVL is used prophylactically only on medium to large varices.

In comparison of nonselective ß-blocker therapy and EVL in the prevention of primary bleeding, both have been found to be effective in reducing primary bleed rate. Moreover, there does not appear to be a mortality benefit between the two. However, some studies report EVL as the cause for bleeding of varices post banding (Toubia & Sanyal, 2008), and therefore EVL is considered the alternate choice after pharmacological therapy with nonselective ß-blocker therapy.

As the final part of management of portal hypertension, patient teaching should include self-monitoring for signs of bleeding. Occult bleeding can happen as a result of portal gastropathy. Patients may notice the passage of dark, tarry stools or melena. Also, bleeding can occur from a less obvious site of varices in portal hypertension, such as rectal varices. Rectal varices can result in passage of maroon (hematochezia), or bright red blood per rectum.

Patients should be encouraged to report increasing fatigue, dizziness, syncope, or chest pain. All of these could be an indication of bleeding. Patient teaching should include instruction on the use of guaiac smear tests. These simple test cards can be prepared at home by placing a smear of stool on each window of the test card and then sending it to the office for evaluation. Also, with each office visit a simple guaiac test can be performed as part of the visit protocol. This will serve as another opportunity to detect occult bleeding.

## Implications for practice

Patients with cirrhosis or those with known portal hypertension from other disease states should be screened for esophageal varices. This is important because esophageal varices are fragile blood vessels with a high propensity to bleed. If no varices are found, routine screening should occur every 3 years. If small varices are found, the frequency is increased to every 2 years. The Child-Pugh score should also be used to evaluate for an increased risk of complications from portal hypertension.

For those patients with medium or large varices, primary prophylaxis should be instituted. This would include use of nonselective ß-blocker therapy to decrease portal hypertension and lessen the likelihood of bleeding. The medications that are recommended are nadolol started at 40 mg daily and increased to obtain a resting pulse rate reduced by 25% or a heart rate of 55 beats per minute. Alternately, propranolol can be started at 40 mg twice daily and increased by 10 mg twice daily to obtain a 20% reduction in resting heart rate. If side effects occur, these medications can be titrated up more slowly in order to give patients more time to adjust.

It should be noted that these patients must remain on lifelong pharmacologic therapy to maintain the beneficial effect of preventing variceal hemorrhage (Turnes et al., 2006). Yet, many times patients with documented esophageal varices are not on ß-blocker therapy. In the study by Wilbur and Sidhu (2005) examining the charts of 106 patients with liver disease who were hospitalized with suspected variceal hemorrhage, none were on ß-blockers. As medical professionals we must do a better job with this treatment protocol; as NPs our background as patient advocates and patient educators will assist us in teaching patients the necessity of this treatment.

Patients started on nonselective ß-blocker therapy must be educated about potential side effects. The most common side effects include hypotension, fatigue, dizziness, impotence, dry mouth and nausea, vomiting, diarrhea, or constipation. If patients are started on lower doses and titrated up slowly, these side effects will usually subside. Patients should understand the effect of the medication and the necessity of treatment. Patient teaching on how to take a radial pulse will help with appropriate titration of medication. This type of educational process will increase medication compliance. If, after educating patients and titrating slowly, the patient is unable or unwilling to continue then the next line therapy should be considered.

As an option for those persons who are unable to tolerate nonselective ß-blocker therapy, contraindicated for nonselective ß-blocker therapy, or noncompliant with the medication regimen, EVL is the second line treatment plan. This is a relatively invasive procedure with the additional concern of sedation and the accompanying risks. Most often multiple EVL procedures are required in order to attain eradication of varices. Still, this is a viable treatment option and can reduce the risk of primary bleeding of esophageal varices.

It is important to remember that nonselective ß-blocker therapy may provide an added benefit of reducing the incidence of bacterial translocation. This process can occur because of the decreased intestinal transit time and the increased bacterial overgrowth that results. Patients with portal hypertension not only have an increased risk of developing esophageal varices, but also an increased risk of ascites. The mere presence of ascites carries the threat of developing spontaneous bacterial peritonitis. Because spontaneous bacterial peritonitis also has a high mortality risk, decreasing the risk is vital.

Patients with cirrhosis should also be taught to self-monitor for other signs of bleeding such as from portal gastropathy or rectal varices. Stools should be monitored for melena, hematochezia, or bright red blood per rectum. Fatigue or dizziness should be reported as these could be signs of occult bleeding rather than a side effect of ß-blockers. Baseline and periodic complete blood counts should be monitored for evidence of bleeding as well as using fecal occult blood tests, such as guaiac smear test, for screening at each office visit. This test can also be prepared by the patient at home and mailed to the office for routine evaluation.

As the NPs caring for these patients we must also be vigilant at reducing further injury to the liver. If the cirrhosis is caused by alcohol, counseling to stop or at the very least decrease intake is imperative. If the damage has not overwhelmed the liver, progression of the disease can be halted or slowed. For those patients who have cirrhosis another important consideration is careful screening of medications. Because many of the medications taken are metabolized via the liver, doses may need to be adjusted or an alternate medication chosen. Through careful assessment, examination, and treatment, these patients can effectively lessen complications and increase their life span.
